# Title of “Ambassador of Clinical Psychology and Psychological Treatment” Awarded to Danutė Gailienė

**DOI:** 10.32872/cpe.7747

**Published:** 2022-09-30

**Authors:** Evaldas Kazlauskas, Andreas Maercker

**Affiliations:** 1 Center for Psychotraumatology, Institute of Psychology, Vilnius University, Vilnius, Lithuania; 2 Department of Psychology, Division Psychopathology and Clinical Intervention, University of Zurich, Zurich, Switzerland

**Keywords:** Danutė Gailienė, psychotraumatology, suicidology, societal impact

## Abstract

The paper presents professional activities and the major works of an ambassador of the European Association of Clinical Psychology and Psychological Treatment (EACLIPT), Prof. Danutė Gailienė. Prof. Gailienė is among the most influential European clinical psychologists who contributed to clinical psychology training, research, and practice in former post-communist East European countries. Her entire career was dedicated to the development of clinical psychology, and through her work, Prof. Gailienė demonstrated how even in an oppressive and politically difficult environment, it is possible to keep the integrity and work up to higher standards.

Ambassador of the European Association of Clinical Psychology and Psychological Treatment (EACLIPT) Prof. Danutė Gailienė was born in 1951 in Lithuania which was occupied by the Soviet Union at a time. In 1969 the first psychology training program was launched at Vilnius University in Lithuania, and she enrolled at the university to study psychology that year. Due to ideological reasons of refusal of any individuality, clinical psychology and psychotherapy were not approved by the Communist Regime (Gailienė, 2000), and the psychology study program was focused on industrial and engineering psychology (Bagdonas et al., 2008) at the time.

However, Danutė Gailienė was very interested in clinical psychology, and since the beginning of her psychology studies, she has aimed to pursue a career as a clinical psychologist. Danutė Gailienė, against the odds, managed to get the position of the first clinical psychologist in a clinical setting in the country during Soviet Regime. Thus, she began to make an outstanding contribution to clinical psychology in the region. She has been the first professor of clinical psychology in the country and was the founder and chair of the clinical psychology program.

**Figure:**
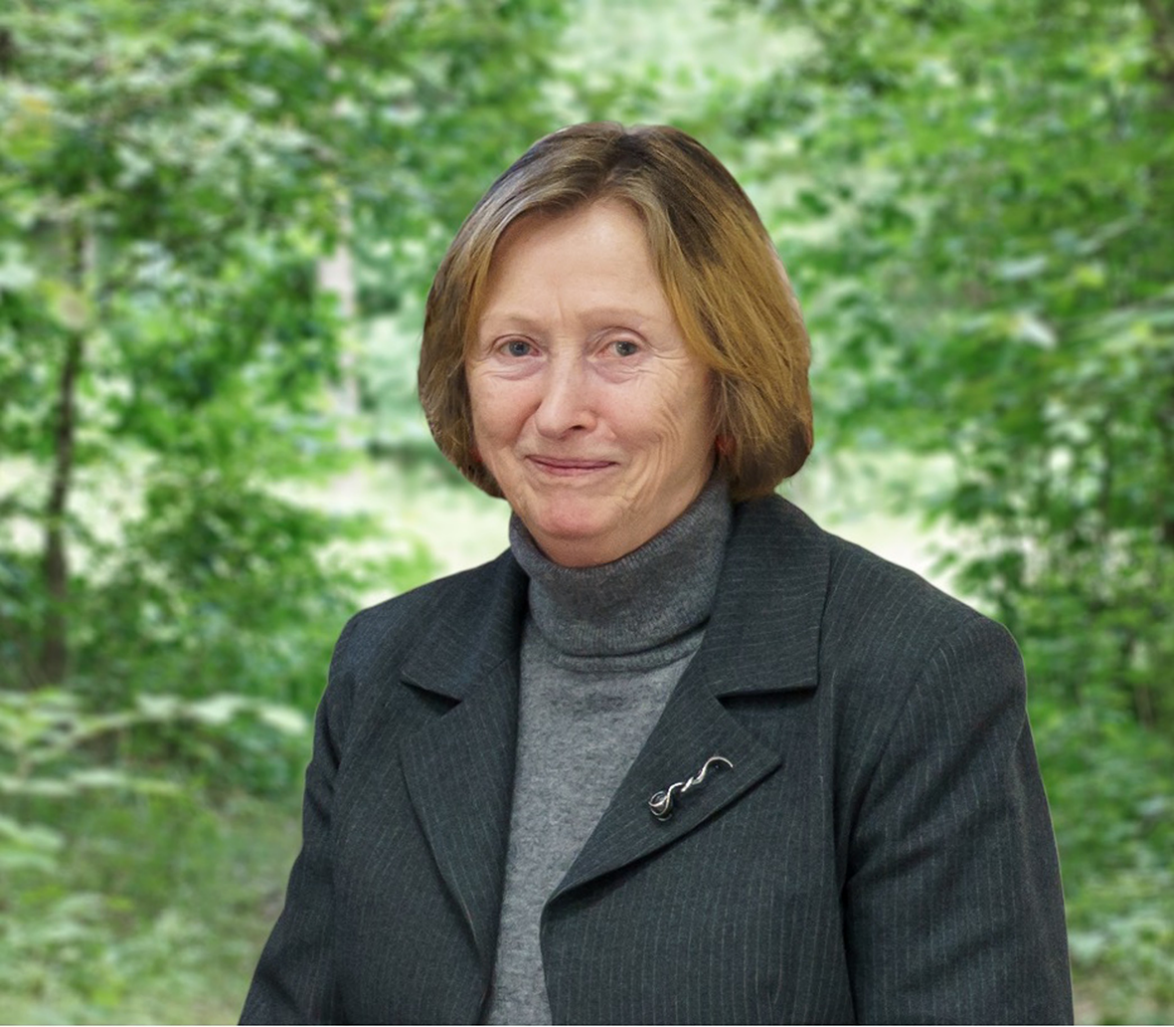
Prof. Danutė Gailienė – Lithuanian psychologist and pioneer of clinical psychology behind 'Iron Curtain.'

## Early Career During Soviet Occupation

Danutė Gailienė graduated from Vilnius University in Lithuania in 1974. The Head of the Psychology Department was Prof. Alfonsas Gučas, who was very supportive of young professionals. Prof. Gučas managed to include a small number of special courses related to clinical or health psychology, even in a very restrictive political situation where political officials in Moscow fully controlled the curriculum. Danutė Gailienė was very interested in clinical psychology during her studies and insistently searched for possibilities to work as a clinical psychologist after obtaining her diploma. However, such positions were not available due to the critical attitude of the Soviet regime towards clinical psychology. Due to her persistence, Danutė Gailienė managed to get a position as a psychologist in one of the psychiatric hospitals in Vilnius and was the first psychologist to work in a psychiatric hospital in the country and the Baltic republics.

Danutė Gailienė was searching for advanced training; however, the possibility of receiving a Ph.D. degree in her preferred area in psychology was not possible due to the mentioned ideological reasons. It required a lot of dedication and hard work, especially to somebody not loyal to the communist party, to receive a Ph.D. Danutė Gailienė worked on her Ph.D. thesis (called 'candidate of sciences' at the time) on cognitive processes in schizophrenia supervised by the internationally famous experimental psychologist, Prof. Bluma Zeigarnik (herself born in Lithuania; discoverer of a psychological cliffhanger effect named after her) from Moscow. At the same time, she was working in Vilnius in a clinical setting, was invited to teach at the university, and raised her three children. Dr. Gailienė received her Ph.D. in psychology from Moscow State University in 1985.

During that time, psychology was highly affected by communist ideology (e.g., the primacy of the ruling party, the material sphere was to be given precedence over the subjective sphere) in the Soviet Union, and the regime was highly oppressive. The psychologist had very restricted or no access to international journals or books. So active and eager to get knowledge, professionals had to find ways for their professional development. A very significant impact on the development of Danutė Gailienė was a visit of Prof. Vytautas Bieliauskas from USA in 1977 (Bieliauskas, 1977), and following his visits. Prof. Bieliauskas was a Lithuanian professor of clinical psychology in the US who managed to come to Lithuania during Soviet occupation and provided training and supervision for a selected group of professionals. The other ways of getting knowledge were Poland and East Germany, which had slightly less restrictive regimes and more access to international professional literature (Leuenberger, 2001). It was also possible to visit Poland and the East German Democratic Republic for training and conferences, and Danutė Gailienė used this opportunity to travel and meet professionals and achieve more specialized knowledge on clinical psychology and psychological treatments.

Not a communist party member and critical of communist party ideology Danutė Gailienė in the 1980s had limited possibilities for an academic or professional career as such professionals were under constant surveillance by the KGB. Having to start her career during Soviet times, which was marked by betrayal, opportunistic loyalty to the communist regime by some of her colleagues who wanted to have a faster and safe career, she has always understood the importance of integrity, a robust value system, and ethical behavior which guided all her professional career.

## The Collapse of the Soviet Union and Career Breakthrough

In the late 1980s, "perestroika" emerged, which was the first signal for the eventual collapse of the Soviet Union. The years of 1988–1990 was a turning point in society in Lithuania and globally. Brave intellectuals, and Danutė Gailienė, among them, participated in peaceful demonstrations against the Soviet Regime and Soviet Occupation. On March 11, 1990, the Lithuanian Parliament declared independence from the Soviet Union.

Almost immediately after the collapse of the Soviet Union, Danutė Gailienė with colleagues interested in clinical psychology (R. Bieliauskaitė, G. Gudaitė, R. Kočiūnas) established the first Department of Clinical and Social Psychology, and the first clinical psychology program was launched in Lithuania (Kazlauskas & Grigutyte, 2020). In the again independent country, Danutė Gailienė could be promoted to a full professor in clinical psychology (2001), was chair of the Department of Clinical (and Social) Psychology (2000-2017). Over the years, she supervised many Ph.D. students who conducted research in clinical psychology and could write their dissertations in Lithuanian.

Prof. Gailienė has been teaching a Clinical Psychology course for undergraduate students, Trauma and Crisis Psychology course in a clinical psychology program (since 2000), and delivering post-diploma training in clinical psychology. Without restrictions to travel abroad, she was a visiting researcher at Munster University in Germany (2003), Antwerp and Gent Universities (2004). Prof. Gailienė was frequently participating in international conferences. Since the start of her career, Prof. Gailienė maintained her clinical practice with at least one day per week meeting clients over decades of her professional activities, and expected her staff at the Department of Clinical Psychology to have an active clinical practice, as an integral part of their professional life.

One of her pioneering works in Lithuania and the region was the first systematic study on suicide prevalence in her country (e.g., Gailienė, 2004a; Gailienė et al., 1995; Gailienė & Ružyte, 1997). Furthermore, she was among the first to study the effects of the communist regime's political oppression in former post-communist countries.

## The Major Works by Danutė Gailienė

Taken together, Danutė Gailienė has been particularly interested in the impact of societal and cultural factors on mental health processes. Her groundbreaking research in suicide prevention was published in her monograph *"They should not have died. Suicide in Lithuania* [*Jie neturėjo mirti. Savižudybės Lietuvoje*]*"* (Gailienė, 1998). This book is fundamental for its first comprehensive analysis of epidemiological data on suicide rates in Lithuania. It analyzes social and cultural factors of a steep increase in around 10 times of suicide rates from the beginning of the 20^th^ century to the last decade of the 20^th^ century in Lithuania, resulting in among the highest in Europe and the World. Prof. Gailienė draws parallels in an increase in suicide rates as an indicator of the public mental health status in response to the social transitions and transformations, primarily associated with devastating effects of long-term political violence and oppression of the Soviet regime. Following an analysis of the suicidal behavior in the country, Prof. Gailienė edited a volume *"Ideas of suicide prevention* [*Savižudybių prevencijos idėjos*]*"* published in 2001. This influential volume included other leading suicidology experts working from Lithuania, Norway, Canada, Slovenia, and Germany on effective suicide prevention programs. She became a widely known suicide researcher in Europe as a result of this research, representing clinical psychology at many expert meetings and congresses across disciplines.

After demonstrating the importance of societal and cultural factors on self-destructive behaviors, Prof. Gailienė made a profound impact in the area of research of political oppression by initiating the first large scale study of survivors of political violence in the country during the Nazi and Communist regimes in particular, former political prisoners and displaced population to the remote areas of Siberia and other areas. The project was initiated in 2000 and was conducted in collaboration with the Lithuanian Genocide and Resistance Research Center. In the course of the research project, a much-acclaimed conference was organized in Vilnius, which focused on the effects of political oppression (Kazlauskas & Zelviene, 2016). As a result of the conference, an important book, *"The Psychology of Extreme Traumatisation: The Aftermath of Political Repression"* was published in Lithuanian in 2004 and English in 2005 (Gailienė, 2004b, 2005). This volume was among the first fundamental works exploring the effects of political violence and oppression in the region of the former Soviet hemisphere by showing how Lithuanian historical trauma and psychotraumatology research should be included in the global agenda of traumatic stress studies.

The next important monograph by Prof. Gailienė was published in Lithuanian *"What they did to us. Lithuanian life in the view of trauma psychology* [*Ką jie mums padarė: Lietuvos gyvenimas trauma psichologijos žvilgsniu*]*"* (Gailienė, 2008). This book provided a deeper view of the impact of the Soviet regime occupation on Lithuanian mental health and is an important contribution to how the general population and professionals could use the theoretical conceptualization and empirical data from a psychotraumatology perspective to discuss complex social issues. The book was published in the context of some nostalgia of the Soviet period in the population and attempts from former communist party leaders and their associates to clean their reputation in stating that they were doing their best in people's interest during the Soviet regime. Prof. Gailienė's book had a significant impact of showing how the communist regime had negative long-term consequences on society (Gailienė, 2008). This work resonated in other countries such as the Baltic countries and Poland, where her name thus became recognized.

A further larger project by a major grant from the European Social Fund resulted in another book both in Lithuanian and in English *"Lithuanian Faces After Translation" Psychological Consequences of Cultural Trauma"* (Gailienė, 2015a, 2015b). It reveals the diversity of the effects of political trauma and the multigenerational impact of prolonged traumatization. A chapter on cultural trauma is the highlight of this book which explores differences and similarities of psychological and cultural trauma based on the Lithuanian historical context (Gailienė, 2015c).

## Final Thoughts

Prof. Danutė Gailienė dedicated her life to the advancement of clinical psychology. Her efforts in pursuing training in clinical psychology and psychological treatments, dissemination of clinical psychology knowledge, assisting patients, teaching clinical psychology at university, training other professionals, establishing a department and clinical psychology program is a clear manifestation of how even under the conditions of an oppressive political regime it was possible to overcome barriers. As an excellent educator, over the years, Prof. Gailienė developed a much praised style of teaching. She received numerous awards for her outstanding work in Lithuania and frequently appeared in national media, commenting on various social and public health issues.

Moreover, Prof. Gailienė has always stressed the importance of the social responsibility of clinical psychologists as professionals. From the perspective of Prof. Gailienė, clinical psychologists must use their knowledge not only to help and treat individual clients but also should be active in social and political life in the country, join professional networks, participate in legislation relevant to psychology and mental health, and be active in the dissemination of knowledge for general population via media. We can conclude that Prof. Gailienė is an outstanding European psychologist. Her personal and professional integrity and dedication to establish the discipline of clinical psychology out of a hostile societal environment can as an ambassador of EACLIPT inspire the future generation of psychologists worldwide.
